# Nomenclatural changes in Onagraceae

**DOI:** 10.3897/phytokeys.145.51139

**Published:** 2020-04-10

**Authors:** Peter C. Hoch, Kanchi Gandhi

**Affiliations:** 1 Missouri Botanical Garden, 4344 Shaw Blvd., St. Louis, Missouri 63110-2291, USA Missouri Botanical Garden St. Louis United States of America; 2 Harvard University Herbaria, 22 Divinity Ave., Cambridge, MA 02138, USA Harvard University Herbaria Cambridge United States of America

**Keywords:** Boisduvalia, Chamaenerion, fireweeds, Ludwigia, nomenclature

## Abstract

A new subspecies and two new combinations are proposed in Onagraceae. Ludwigia glandulosa Walter subsp. brachycarpa C.-I Peng, **subsp. nov**. is morphologically distinct from the typical subspecies, with smaller capsules and leaves, different seed coat, and a restricted distribution. Epilobium sect. Pachydium (Fischer & C. A. Meyer) Hoch & K. Gandhi, **comb. nov**. refers to a distinctive group of species formerly known as *Boisduvalia* Spach and as Epilobium sect. Boisduvalia (Spach) Hoch & P. H. Raven. And *Chamaenerion speciosum* (Decaisne) Hoch & K. Gandhi, **comb. nov**. is proposed for a distinctive Himalayan species originally described in *Epilobium*.

## Introduction

The plant family Onagraceae is known in considerable detail as a result of modern monographic studies of almost the entire family and numerous comparative morphological analyses, summarized in Wagner et al. (2007). Recent phylogenetic analyses (especially Levin et al. 2003, 2004) provided much insight into the relationships in the family and necessitated many changes in the classification. Most of these changes were included in Wagner et al., but recent work revealed the need for several additional nomenclatural changes.

## Methods

This contribution is the result of careful nomenclatural review by K. Gandhi of the treatment of Onagraceae by WL Wagner and PC Hoch for the Flora of North America. Gandhi detected several nomenclatural problems, which are corrected by the following changes.

## Taxonomic treatment

*Ludwigia* L., a pan-subtropical genus of 82 species, forms a strongly monophyletic lineage sister to the rest of Onagraceae (e.g., Levin et al. 2003, 2004). Recent molecular analysis (Liu et al. 2017) has challenged the complex sectional classification of *Ludwigia* (22 sections), but full resolution awaits more detailed analysis. One strongly monophyletic clade is section Isnardia (L.) W. L. Wagner & Hoch, a group of 19 species centered in southeastern North America (Liu et al. in press) and formerly treated as sections *Dantia* (DeCandolle) Munz (Peng et al. 2005) and *Microcarpium* Munz (Peng 1989; Wagner et al. 2007). While reviewing the treatment of Onagraceae for the Flora of North America, one of us (KG) noticed an error in the treatment of *L. glandulosa* Walter, a widespread species of section Isnardia. Peng (1986) initially published L. glandulosa subsp. brachycarpa (Torrey & A. Gray) C. Peng as a comb. nov., based on *L. cylindrica* Elliott β. *brachycarpa* Torrey & A. Gray, but that latter name was a comb. nov. based on *Jussiaea brachycarpa* Lam., which Peng considered to be a synonym of L. glandulosa subsp. glandulosa. Therefore, L. glandulosa subsp. brachycarpa was not a comb. nov., as Peng proposed, but instead a subsp. nov. However, it was invalid since it lacked a diagnosis/description in Latin, as required at that time, and designation of a type specimen. Here we correct the mistake, and validate the name with a type and description. Unfortunately, our colleague Ching-I Peng died in 2018, but since his original intention was clear and he provided the name and description (Peng 1986, 1989), with this authorship we honor his enormous contributions to our understanding of *Ludwigia*.

### Ludwigia glandulosa Walter subsp. brachycarpa

Taxon classificationPlantaeMyrtalesOnagraceae

C.-I Peng subsp. nov.

2D8175C7-8DB2-50E7-A84D-E0BAFF94F255

urn:lsid:ipni.org:names:77209334-1

#### Diagnosis.

Ludwigia glandulosa subsp. brachycarpa differs from typical *L. glandulosa* in its smaller stature, 10–55(–90) cm (vs. (20–)40–80(–100) cm); narrower leaf blades, 0.3–0.5(–1) cm (vs. 0.3–2.1 cm); shorter sepals, 1.1–1.9 mm (vs. 1.3–2.3 cm); smaller capsules, 2–5 × 1.3–2 mm (vs. (4–)5–8(–9) × 1.6–2(–3) mm); and seed surface cells elongate transversely to length (vs. surface cells elongate parallel to length).

#### Type material.

**USA, Louisiana**, Cameron Parish, 3.2 km W of junction State Highways 82 and 27, 29°48.62'N, 93°08.25'W, 1 m, 16 August 1980, *C.-I Peng, W. Peng and J. Chen 4367* (holotype: MO 2806683); see fig. 42 in Peng (1989).

#### Description.

***Stems*** rarely reddish green, 10–55(–90) cm. ***Leaves***: petiole 0–1 cm, blades linear-elliptic to linear, sometimes very narrowly elliptic, those on main axis 3–5(–7) × 0.3–0.5(–1) cm, those on branches 0.8–3.6 × 0.2–0.3(–0.8) cm. ***Inflorescences***: bracteoles attached at base of ovary, 0.4–0.8 × 0.1–0.2 mm. ***Flowers***: sepals 1.1–1.9 × 1–1.8 mm, apex acute or short-acuminate; nectary disc obscurely, minutely papillose; style 0.4–0.8 mm, stigma 0.2–0.3 mm diam. ***Capsules*** obscurely 4-angled, 2–5 × 1.3–2 mm, pedicel 0–0.2 mm. ***Seeds*** 0.6–0.8 × 0.3–0.4 mm, surface cells elongate transversely to seed length. ***Chromosome number***: *n* = 16.

#### Phenology.

Flowering and fruiting April to November.

#### Etymology.

The subspecific epithet ‘*brachycarpum*’ refers to the short capsules.

#### Distribution and habitat.

Ludwigia glandulosa subsp. brachycarpa is endemic to the US Gulf Coast from southwestern Louisiana to Nueces County, Texas, and more sporadically northward in eastern Texas to south-central Oklahoma. This distribution is at the extreme southwestern edge of that for subsp. glandulosum, which grows from Texas and Oklahoma east to Virginia and Florida and north to southern Missouri, Illinois, and Indiana (Peng 1989). Although they overlap in part, the two taxa are only rarely locally sympatric. Ludwigia glandulosa subsp. brachycarpa grows in ditches, low meadows, coastal prairies, seeps in sandy woods, moist sinkholes in granite outcrops, old clay fields at an elevation of 0–200 m.

##### New combinations

*Epilobium* L. is the largest genus in the family Onagraceae; its 165 species are widely distributed in cool or cold regions of the world, with a center of diversity in western North America (Wagner et al. 2007). A group of annual species with affinities to *Epilobium* but considered distinct because they lack seed comas was historically segregated as *Boisduvalia* Spach (Raven 1976). However, molecular and other evidence (Hoch and Raven 1992; Baum et al. 1994; Wagner et al. 2007) unequivocally place *Boisduvalia* within *Epilobium* as two non-monophyletic sections. Epilobium sect. Epilobiopsis (Spegazzini) Lievens, Hoch & PH Raven is a group of two species characterized by tough, tardily dehiscing capsules, seeds in two rows per locule, and a chromosome number of *n* = 15; and E. sect. Boisduvalia (Spach) Hoch & P. H. Raven is a group of four species characterized by friable, readily dehiscing capsules, seeds in one row per locule, and chromosome numbers of *n* = 9, 10, 19. In naming this latter section, however, we overlooked an earlier name at the sectional level, one of two proposed for this group by Fischer and Meyer (1836), who treated the group as part of *Oenothera* L. due to the absence of seed comas. Therefore, a new combination is required.

### Epilobium Linnaeus sect. Pachydium

Taxon classificationPlantaeMyrtalesOnagraceae

(Fischer & C.A. Meyer) Hoch & K. Gandhi comb. nov.

7DCD14C0-9B83-5487-BC3D-F2C9A6ACDCD1

urn:lsid:ipni.org:names:77209335-1

#### Basionym.

Oenothera sect. Pachydium Fischer & C.A. Meyer, Ind. sem. hort. petrop. 2: 45. 1836 [“1835”]. *Boisduvalia* [unranked] *Pachydium* (Fischer & C.A. Meyer) Endlicher, Gen. pl. 1191. 1840. B. subg. Pachydium (Fischer & C.A. Meyer) Reichenbach, Deut. Bot. Herb.-Buch. 170. 1841; Boisduvalia sect. Pachydium (Fischer & C.A. Meyer) Munz, N. Amer. Flora 5, II: 228.

#### Type.

*Oenothera densiflora* Lindley [= *Epilobium densiflorum* (Lindley) Hoch & P.H. Raven].

The distinctive, circumboreal/ circumpolar group commonly known as fireweeds has been treated either as a section of *Epilobium* (Haussknecht 1884; Raven 1976; Chen et al. 1992) or as the separate genus *Chamaenerion* Séguier. Although the two groups share the distinctive comose seeds, several floral features, and a base chromosome number of *x* = 18, *Chamaenerion* differs from *Epilobium* in having leaves nearly always spirally arranged, rarely subopposite or verticillate near stem base (vs. opposite at least on proximal stem); lack of a floral tube (vs. more or less distinct floral tube); flowers slightly zygomorphic with subequal stamens that are erect, then deflexed, and styles that are deflexed, then erect (vs. actinomorphic with erect stamens in two series and erect styles); petals entire (vs. emarginate); and pollen shed in monads (vs. tetrads) (Wagner et al. 2007). Recent molecular analyses (Baum et al. 1994; Levin et al. 2004) also demonstrated that the fireweeds form a strongly supported clade sister to the rest of *Epilobium* (Wagner et al. 2007).

Holub (1972) argued that the correct name for the fireweeds at the generic level should be *Chamerion* (Raf.) Raf. ex Holub, not *Chamaenerion*, which he argued was illegitimate. However, as noted in personal correspondence between KG and Ulf Eliasson in 2009, and summarized by Sennikov (2011), clarifications in the botanical code and in the lectotypification of *Chamaenerion* and *Epilobium* negate Holub’s analysis, and Sennikov concluded that the correct and valid name for the fireweeds at the generic level is *Chamaenerion* Séguier.

All but one of the eight species recognized in this genus have been treated at some point as species in *Chamaenerion*, the only exception being a species described in *Epilobium* that is endemic to the Himalayan region from Kashmir to Nepal and Xizang (Tibet), China (Chen et al. 1992), for which the following new combination is provided:

### Chamaenerion speciosum

Taxon classificationPlantaeMyrtalesOnagraceae

(Decaisne) Hoch & K. Gandhi comb. nov.

A5E87B4C-B74B-58C4-8986-4F479FF599E0

urn:lsid:ipni.org:names:77209336-1

#### Basionym.

*Epilobium speciosum* Decaisne, Voy. Ind. (Jacquemont) 4: 57, t. 69 [Apr 1835 – Dec 1844]. Epilobium latifolium L. subsp. speciosum (Decne.) P. H. Raven, Bull. Brit. Mus. (Nat. Hist.), Bot. 2(12): 349. 1962. *Chamerion speciosum* (Decne.) Holub, Folia Geobot. Phytotax. 7: 86. 1972; non *Chamaenerion speciosum* Lodd. ex Steudel, Nomencl. Bot. ed. 2, 1: 343. 1840, pro syn.

#### Type material.

**India. Himachal Pradesh**, near Yurpo, 3800m 1830–1831, *V. Jacquemont 1739* (***holotype***: P; ***isotypes***: G, K).

## Supplementary Material

XML Treatment for Ludwigia glandulosa Walter subsp. brachycarpa

XML Treatment for Epilobium Linnaeus sect. Pachydium

XML Treatment for Chamaenerion speciosum
